# Surgical management of sacral meningeal cysts by obstructing the communicating holes with muscle graft

**DOI:** 10.1186/s12891-019-2998-x

**Published:** 2019-12-30

**Authors:** Kai Yang, Huiren Tao, Chaoshuai Feng, Jiawei Xu, Chunguang Duan, Weizhou Yang, Wei Su, Huan Li, Haopeng Li

**Affiliations:** 1 Department of Orthopaedics, Second Affiliated Hospital of Xi’an Jiaotong University, 157th West Fifth Road, Xi’an, 710004 Shaanxi Province China; 2 Department of Orthopaedics, Shenzhen University General Hospital, 1098th Xueyuan Road of Xili University Town, Shenzhen, 518055 Guangdong Province China; 3 Department of Orthopaedics, First Affiliated Hospital of Xi’an Jiaotong University, 277th Yanta West Road, Xi’an, 710061 Shaanxi Province China; 4 Department of Cardiology, Xijing Hospital, Military Medical University of PLA Airforce (Former Fourth Military Medical University), 127th Changle West Road, Xi’an, 710032 Shaanxi Province China

**Keywords:** Sacral meningeal cyst, Communicating hole, Muscle graft, Surgical management

## Abstract

**Background:**

The surgical indication and treatment of sacral meningeal cyst have not been well established and current methods are usually accompanied by complications and recurrence. The aim of this study is to discuss the treatment of symptomatic sacral meningeal cyst, by investigating the surgical results of our surgically treated patients, and minimize the complications and recurrence.

**Methods:**

We retrospectively reviewed all patients with symptomatic sacral meningeal cysts who were surgically treated by a single surgeon in the same institution from 2002 to 2017. All patients underwent the same operation by incising the cyst wall and obstructing the communicating hole with muscle graft, while the cyst wall was left untreated instead of resected or imbricated. The obstruction was verified by doing a Valsalva-like maneuver. The preoperative symptoms and signs, and the outcomes at most recent follow-up were rated and compared by Neurological Scoring System.

**Results:**

A total of 18 patients (7 male patients and 11 female patients, average age 42.3 years) were followed up for an average of 51.7 months. All patients had communicating holes linking the cysts and the dural sacs. The average preoperative neurological score was 19.7 ± 2.2, and it was improved to 23.2 ± 2.8 at the most recent follow-up (*p* < 0.01).

**Conclusions:**

The sacral meningeal cyst originated from the communication with the dural sac. Surgical treatment of symptomatic sacral meningeal cysts can yield a long-term resolution of the appropriately selected patient’s symptoms. Obstructing the communicating hole with muscle graft is an effective and simple method to obliterate the cyst. The incised cyst wall can be left untreated instead of resected or imbricated.

## Background

The cyst located within the sacral spinal canal, known as the sacral meningeal cyst, was well documented in the literature. It was first described by Tarlov in 1938 as an incidental finding at autopsy [1]. About 1.5 to 4.6% of magnetic resonance imaging (MRI) examinations performed for evaluation of low back pain revealed sacral meningeal cysts. The majority of them were asymptomatic and in about 20% of sacral meningeal cysts, seen on MRI, patients had complaints that can be associated with these cysts [2, 3].

Several authors have reported varying outcomes with surgical treatment of the sacral meningeal cysts [4–7]. Nevertheless, various surgical complications were reported and recurrence of the cysts still commonly bothered patients after surgery [5, 8, 9]. Therefore, controversy still exists regarding the surgical indications, operative techniques, and outcomes of surgery. We therefore retrospectively reviewed our surgically treated patients with sacral meningeal cysts, with emphasis on the surgical outcomes and techniques aiming to avoid surgical complications and recurrence.

## Methods

### Patients

Between 2002 and 2017, 18 patients (7 male, 11 female) ranging in age from 20 to 70 years (mean, 42.3 years) with symptomatic sacral meningeal cysts were treated surgically by the senior author (H.T.). The diagnoses of the sacral meningeal cyst were confirmed by MRI in all patients. The medical reports and images were reviewed, as well as the numbers, sizes and locations of the cysts. All procedures performed in this study were in accordance with the ethical standards of the institutional and national research committee and with the 1964 Helsinki declaration and its later amendments or comparable ethical standards. This study was approved by the Institutional Review Board of Second Affiliated Hospital of Xi’an Jiaotong University (No. XJTU1AF2018LSK-78) and informed consents were obtained from all individual participants included in the study.

Only those patients who met following criteria received surgery: 1) clinically, the patient had neurological signs and symptoms, including S1 radicular symptoms (S1 radicular pain, leg numbness or weakness, neurogenic claudication), or sacral plexus disorder (bowel and bladder dysfunction, sexual impotence, perineal/perianal pain). 2) MRI confirmed the existence of the cyst. 3) excluding other causes, such as lumbar disc herniation or spinal stenosis.

The preoperative symptoms and signs, and the outcomes at most recent follow-up were rated by the Neurological Scoring System, which was designed for clinical evaluation of patients with spinal lesions [10]. This scale includes five items (pain, sensory deficits, motor weakness, gait, sphincter function), for each a score between 0 and 5 is given. Five scores for each item and a total of 25 scores indicate a normal neurological status, and the less the score, the poorer the status.

### Surgical technique

Under general or epidural anesthesia, the patient was put in a prone position. After incision of the skin and fascia, subperiosteal dissection of the paravertebral muscle was done with extreme care not to intrude into the sacral canal through the thinned sacral roof, which had been eroded by the cyst. Sacral laminectomy was done meticulously to preserve the integrity of the cysts. The cyst was then fenestrated at its thinnest site using a scalpel to drain off the fluid, and its inner surface was carefully examined. After fenestration of the cyst wall, as long a longitudinal incision as possible was made on the cyst wall to get the widest exposure for convenient inspection of the interior and closure of the communicating hole.

**Fig. 1:**
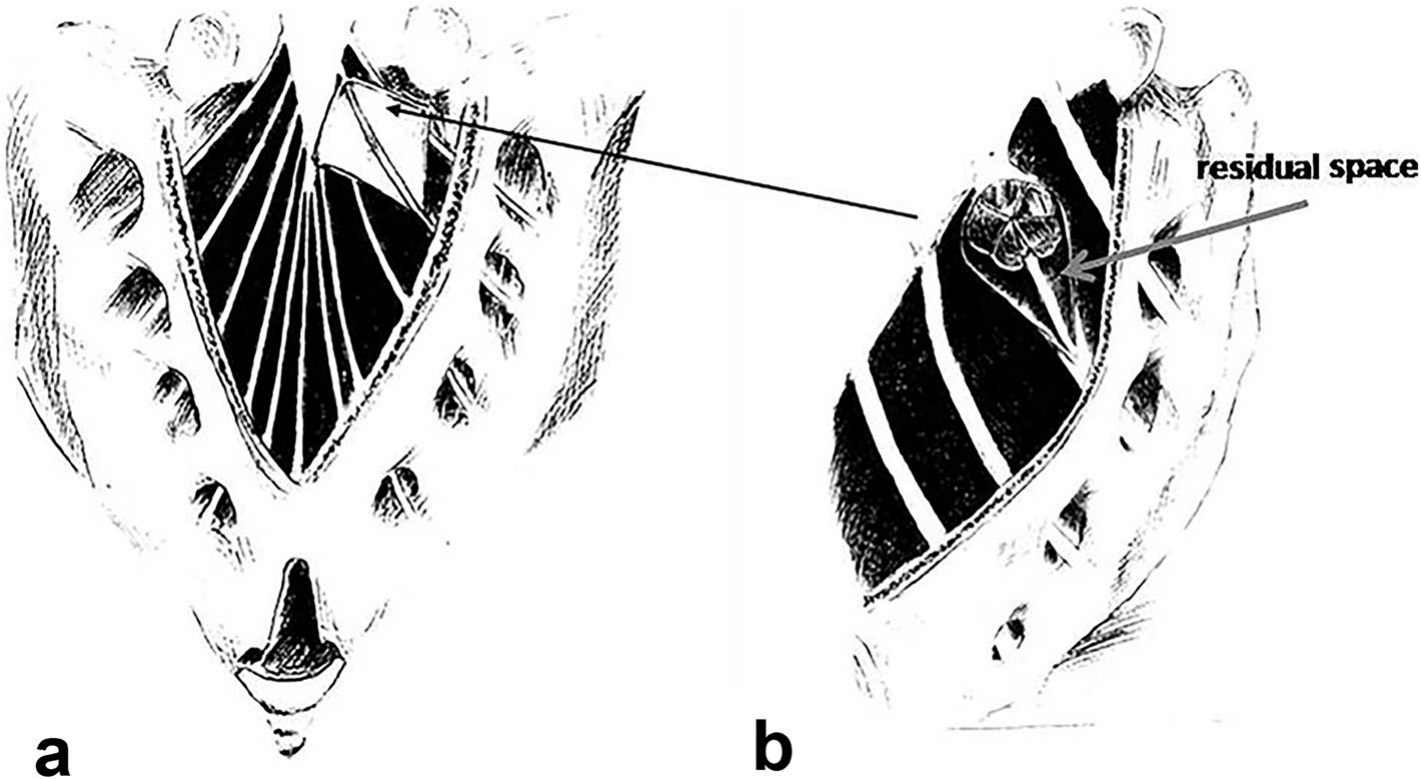
Illustration of surgical technique: (**a**) fenestration and incision of the cyst wall; (**b**) an appropriate block of muscle is used to plug the hole and fixed by a purse-string suture

For the purpose of obstructing the communicating hole to prevent the recurrence of the cyst and postoperative cerebrospinal fluid (CSF) leakage, an appropriate block of muscle was used to plug the hole and was fixed by purse-string suture using 4/0 sutures (Fig. 1). The muscle graft was firmly against the surroundings of the communicating hole, making the closure watertight. In addition, this was verified by doing a Valsalva-like maneuver (pressuring the patient’s abdomen), during which time there should be no CSF flowing into the cyst.

After the communicating hole being blocked, the incised cyst wall was left untreated instead of resected or imbricated. The residual space was filled with gelfoam and/or fibrin glue, or a local pedicled sacrospinalis muscle flap for temporary equalization of the pressure between the cavity and dural sac. Then a closed non-pressure drainage was inserted into the cavity and the wound was closed.

### Postoperative care

After surgery, the drainage was removed when the amount of drain decreased to less than 100 ml, which was usually 24–72 h after surgery. However, an excessive amount of drain indicated failure to block the communicating hole, which might lead to recurrence in the future.

### Statistical analysis

The neurological scores were given as mean value and standard deviation. Statistical analysis was conducted using SPSS software for Windows (version 23.0; IBM Corporation. Armonk, New York, U.S.). Neurological scores before the operation and at most recent follow-up were compared by Wilcoxon signed rank test. The significance level was set at *p* < 0.05.

## Results

### Surgical findings

**Fig. 2:**
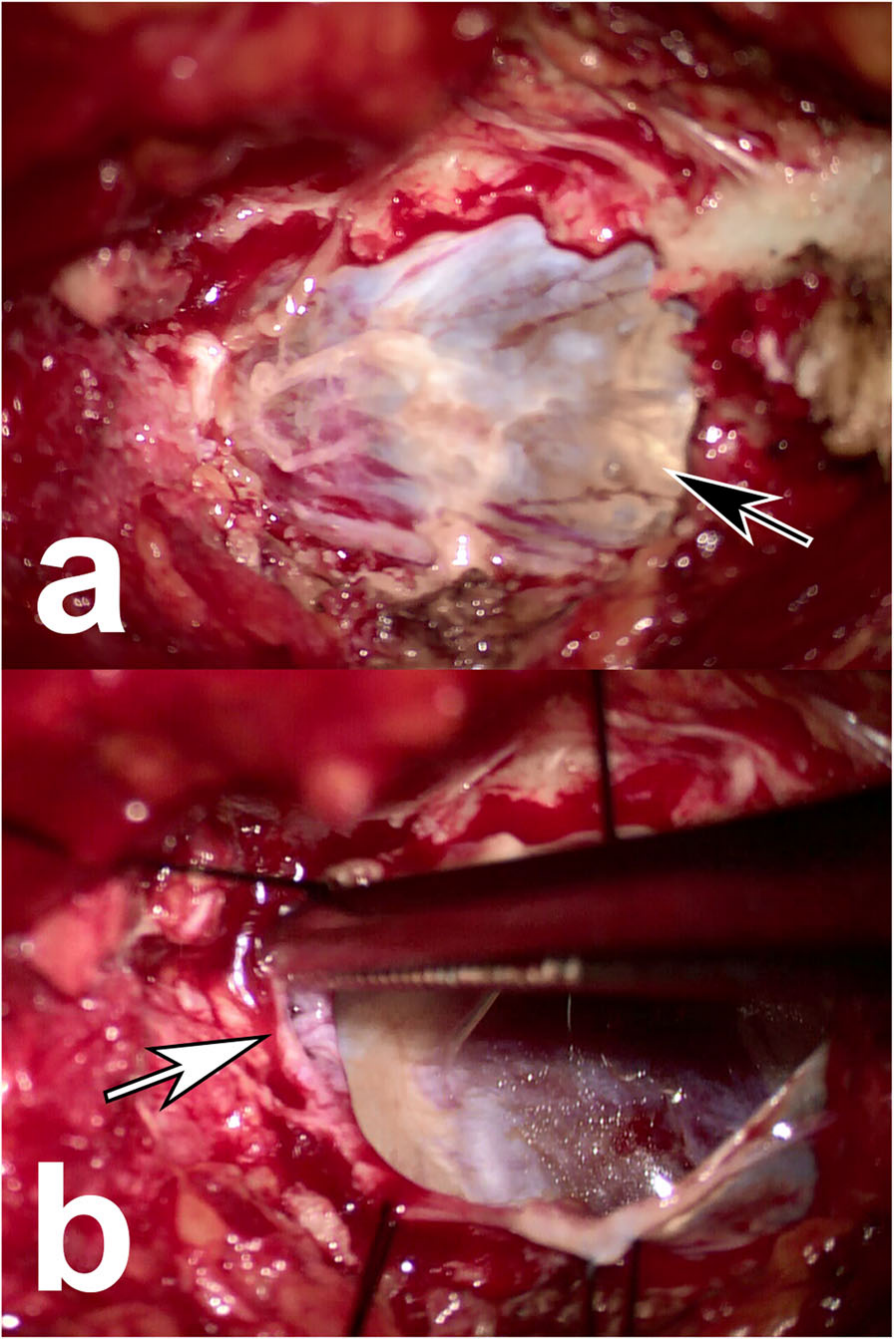
Intra-operative photos of the sacral meningeal cyst: (**a**) the semitransparent cyst wall (black arrow); (**b**) the communicating hole linking the cyst and the dural sac (white arrow)

**Fig. 3:**
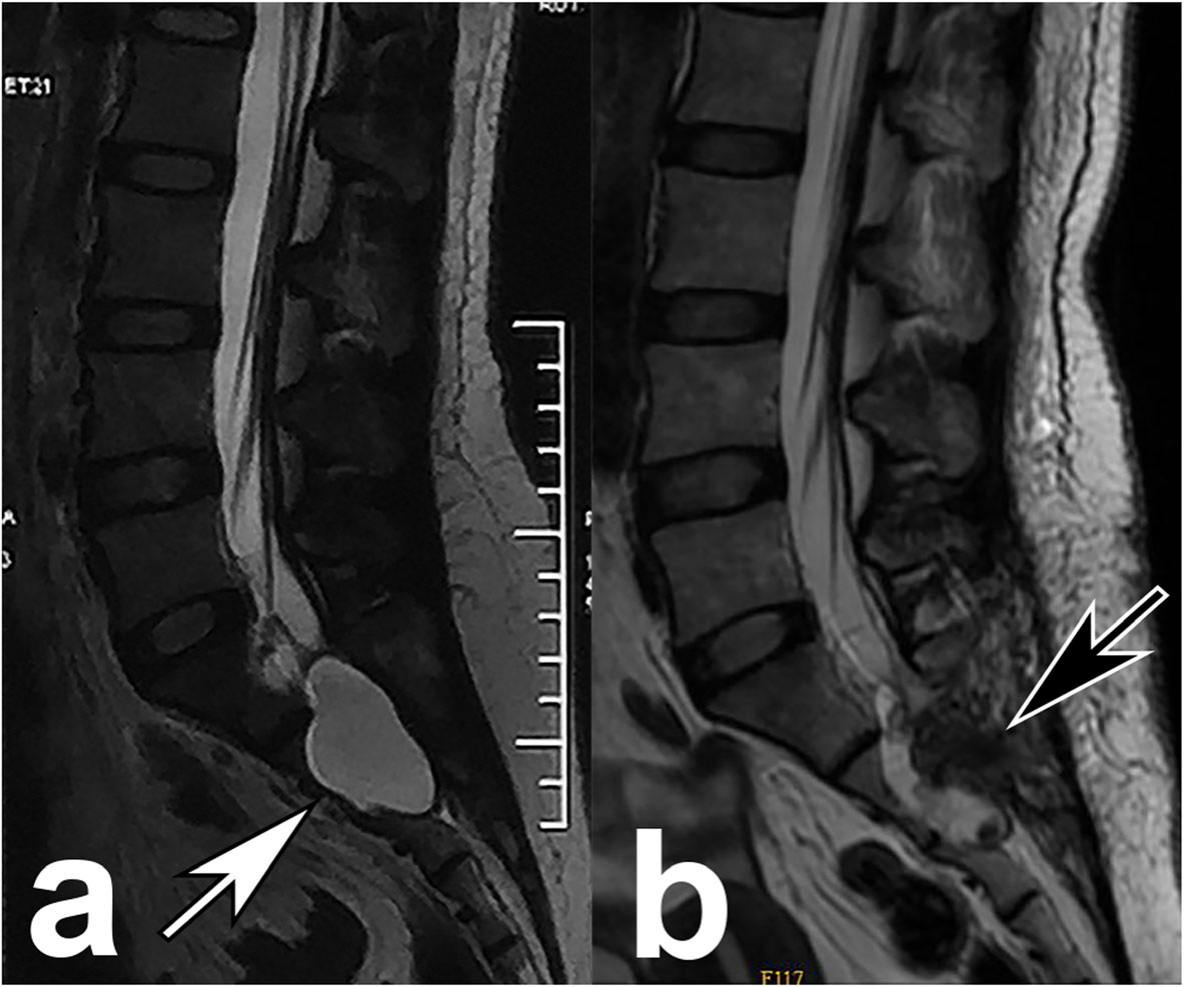
Magnetic resonance images before and after the operation: (**a**) preoperative image shows a sacral meningeal cyst at S1–3 level; (**b**) the image taken 2 days after surgery shows the obliteration of the cyst and the muscle graft (black arrow)

**Fig. 4:**
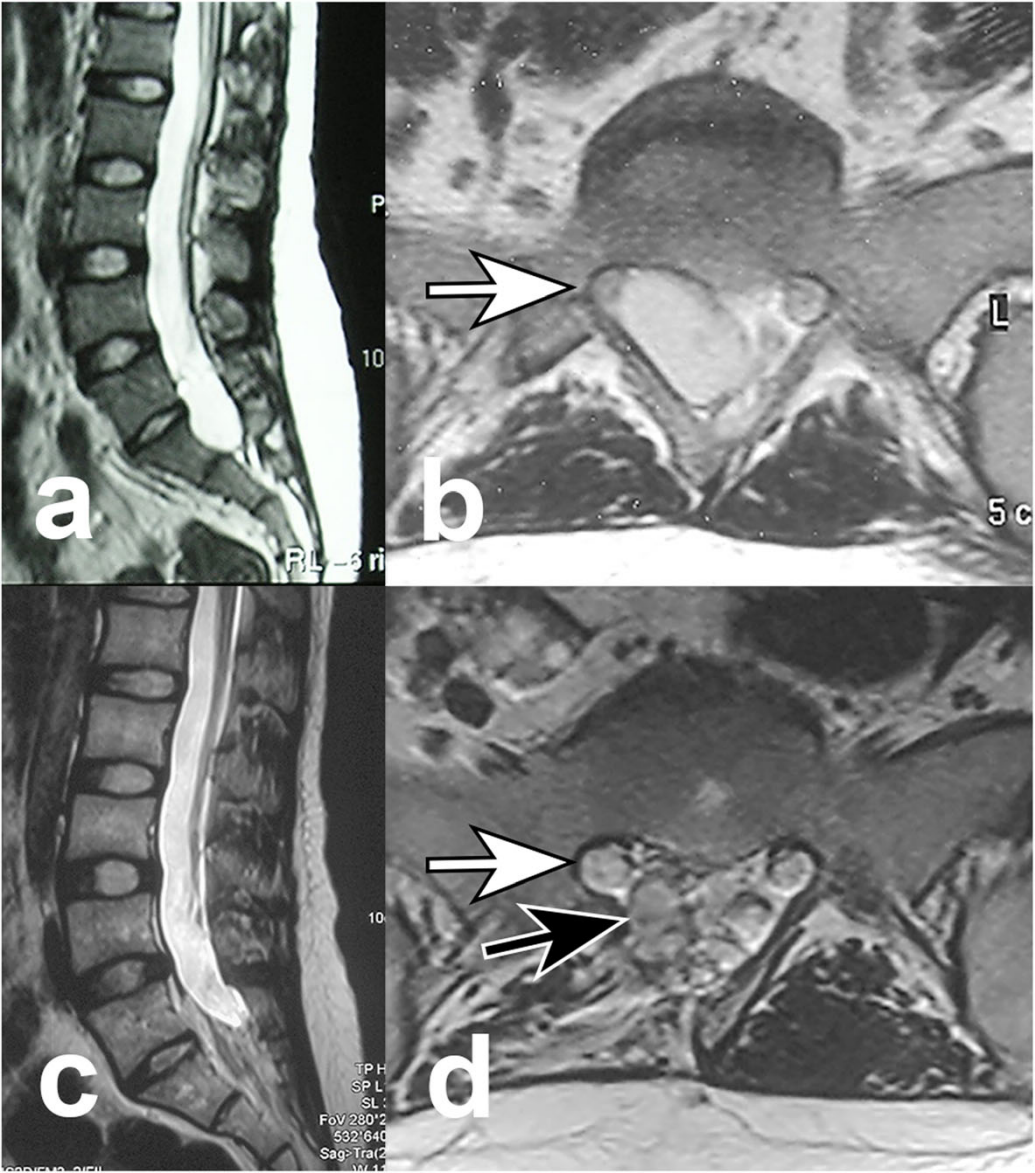
Preoperative sagittal magnetic resonance image (**a**) shows a sacral meningeal cyst at S1–2 level, and transverse image (**b**) shows cystic lesion compressing the right S1 nerve root (white arrow); Taken 64 months after surgery, sagittal image (**c**) shows obliteration of the cyst, transverse image (**d**) shows decompressed right S1 nerve root (white arrow) and stable muscle graft (black arrow)


After bony decompression, semitransparent and highly tensioned cysts were exposed (Fig. 2a), bearing on and eroding the bony wall. All patients had communicating holes linking the cysts and the dural sacs (Fig. 2b). When having the patient perform a Valsalva-like maneuver to increase the abdominal pressure, the CSF would flow into the cyst through the hole (see Additional file 1). Muscle blocks were grafted to the holes in all patients (Fig. 3). Figure 4 shows preoperative and postoperative MRI representing the disappearance of the cyst, decompression of the sacral nerve root and stable muscle graft in the most recent follow-up.

**Additional file 1: Video S1.** Cerebrospinal fluid through communicating hole. A video shows that the cerebrospinal fluid flow through the communicating hole before the obstruction, the flow can be triggered by a Valsalva-like maneuver (pressuring the patient’s abdomen).


Of the 18 patients, fifteen patients presented with sacral meningeal cysts which contained nerve root fibers, three patients had sacral meningeal cysts without nerve root fibers in the cyst. The characteristics of the cysts were shown in Table 1.

**Table 1:** Demographic data and morphological features of the cysts

Patient No.	Age (yr)/Sex	Cyst location	Number of cysts	Maximum size (cm)	Intracystic nerve
1	52/F	S2–3	2	2.5	No
2	35/M	S2–4	1	2	Yes
3	36/M	S1–3	2	5	Yes
4	34/F	S2–3	1	5	Yes
5	49/F	S2	1	2	Yes
6	47/M	S2	2	3	Yes
7	36/M	S1–3	3	3	Yes
8	20/F	S1	1	6	Yes
9	42/M	S1–3	1	8	Yes
10	70/F	S2	1	1.5	Yes
11	31/M	S1	1	5	Yes
12	51/F	S2	4	4	Yes
13	40/F	S2	1	1.8	Yes
14	66/F	S1–2	2	5	Yes
15	35/M	S2	1	3	Yes
16	31/F	S2	1	4	No
17	51/F	S1–3	1	6	Yes
18	36/F	S2	1	4	No

### Surgical outcomes

**Fig. 5:**
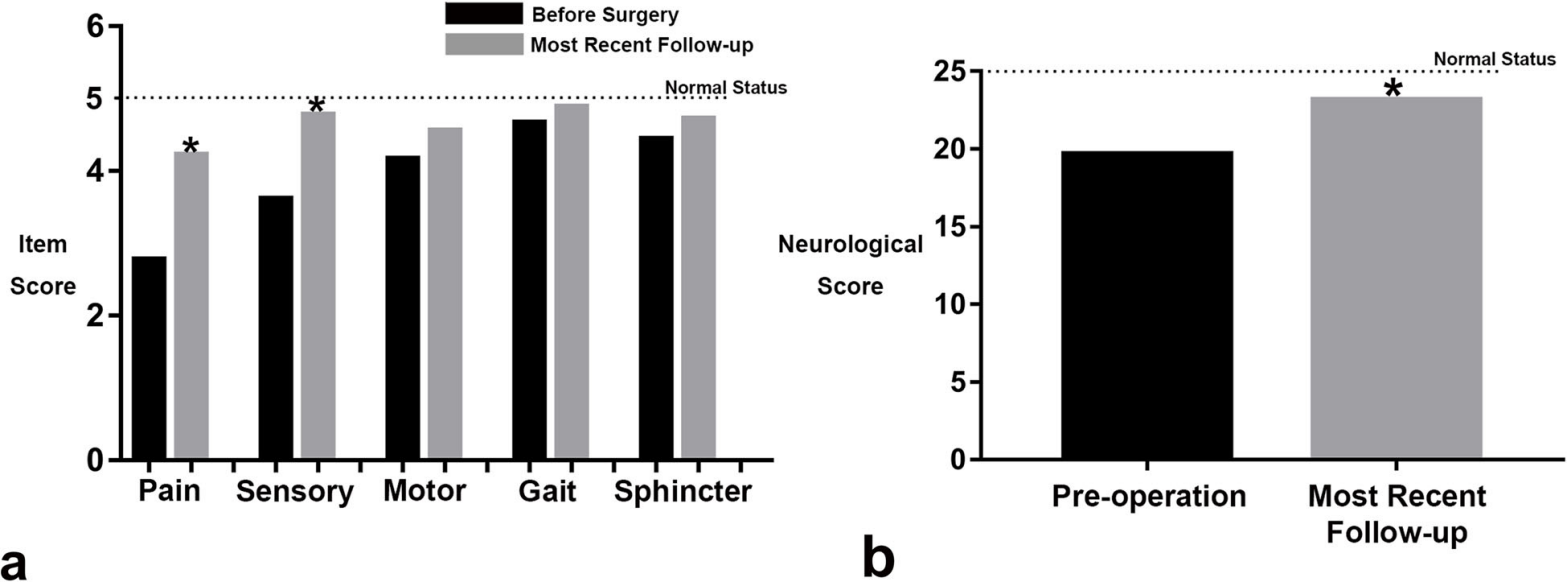
Average neurological scores before surgery (black column) and at most recent follow-up (gray column) for each item (**a**) and in total (**b**), asterisks indicate that the differences are statistically significant (*p* < 0.05)

The patients were followed up for an average of 51.7 months (range, 10–158 months). All the preoperative symptoms and neurological sores before surgery and at the most recent follow-up are listed in Table 2. The overall neurological status of the patients improved significantly. The average preoperative neurological score was 19.7 ± 2.2, and it was improved to 23.2 ± 2.8 at most recent follow-up (*p* < 0.01). During the postoperative recovery, local pain and radicular symptoms were most likely to improve or disappear after surgery, and residual lumbosacral pain was the most common postoperative complaint. For each item of the Neurological Scoring System, the improvement of pain (preoperative 2.8 ± 0.8 improved to 4.2 ± 0.8, *p* = 0.002) and sensory function (preoperative 3.6 ± 1.2 improved to 4.8 ± 0.6, *p* = 0.06) was statistically significant, while motor, gait and sphincter function improved slightly, although not statistically significant (*p* > 0.05) (Fig. 5).

**Table 2:** Symptoms and outcomes

No.	Preoperative symptoms	Follow-up (mon)	Preoperative score	Postoperative score
1	lumbosacral pain radiating to right leg	24	22	25
2	lumbosacral pain radiating to both legs, bowel dysfunction, urinary dysfunction, perineal hypoesthesia, sexual dysfunction	63	18	25
3	lumbosacral pain, numbness in perineum and both legs	30	21	25
4	lumbosacral pain, urinary dysfunction, perineal numbness	10	17	25
5	lumbosacral pain and perineal pain, discomfort in both legs	156	19	25
6	lumbosacral pain radiating to left leg	58	22	25
7	lumbosacral pain and perineal pain, numbness and weakness in both legs	26	18	25
8	lumbosacral pain, numbness and pain in right leg	64	21	24
9	lumbosacral pain radiating to right leg, perineal hypoesthesia	28	21	24
10	lumbosacral pain, radicular pain in both legs	26	20	23
11	lumbosacral pain, numbness in perineum and both legs	12	22	24
12	lumbosacral pain radiating to left leg	158	23	25
13	lumbosacral pain radiating to left leg, perineal hypoesthesia	49	20	24
14	lumbosacral pain radiating to right leg, claudication	26	17	24
15	Discomfort in right leg, paralysed flexor hallucis longus muscle	19	18	19
16	lumbosacral pain radiating to both legs, perianal pain, urinary dysfunction, bowel dysfunction	24	21	21
17	lumbosacral pain radiating to right leg, numbness and weakness in both leg, urinary dysfunction	13	15	15
18	lumbosacral pain, urinary dysfunction, pain and numbness in both legs	144	19	19

Three patients had a recurrence of the cyst, as confirmed by MRI. The recurrence, in one of them (case 16) manifested as persistent perianal pain in addition to deterioration of the lumbosacral pain at 6 months postoperatively, despite the improvement in bowel and bladder function. There was no significant improvement of clinical symptoms in another case (case 17) with recurrence. Another recurrence happened at 1 year after surgery (case 12), and all symptoms were resolved after the second operation. The patient had 4 cysts before the first surgery, three of them were operated while the other one was too small to be treated. During the second surgery, the three operated cysts were found to be the same as that after the first operation with muscularized obstruction in the communicating holes, and significant expansion of the previously untreated cyst was also found. After treating this cyst with the same method, the patient had relief of all symptoms up to the most recent follow-up (158 months).

### Complications

Two patients had epidermis erosion at the distal end of the incision, which healed after repeated dressing changes and physiotherapy. There was no postoperative neurological deterioration or CSF leakage.

## Discussion

### Pathophysiology

In 1964, Holt and Yates [11] found that the cyst cavity had a clear and patent canal that communicated with reticular subarachnoid space, which was supported by myelographic investigation. Many authors subsequently reported on the existence of such valve-like communicating holes [4, 6, 7]. It is believed that a congenital defect or weakness of the dura allowed herniation of the arachnoid under the hydrostatic pressure of the CSF [12–14].

In the current series, communicating holes were found in all the patients. During the surgery, CSF flow through the communicating holes was found, which can be triggered by increasing the abdominal pressure using a Valsalva-like maneuver. This fact further supports following hypothesis: the CSF flows into the congenital pouch (cyst) via the communicating hole when the abdominal pressure is increased or with the pulsation of artery, but the ball-valve mechanism does not allow the CSF to flow back to the thecal sac as the communicating hole is one-way open to it, and consequently the cyst is gradually distended [2, 15].

### Surgical indications

Since not all of the sacral meningeal cysts are symptomatic, it is important to identify the cysts which are responsible for the clinical complaints, which is pivotal to successful treatment. Voyadzis [16] suggested that patients with neurological symptoms that are anatomically related to Tarlov cysts greater than 1.5 cm in diameter could benefit from surgical treatment. Tanaka [7] suggested that a positive filling defect in myelography may be an indicator of good treatment outcomes. Up to now, there have been no explicit criteria whereby to determine the indications of surgery for sacral meningeal cysts.

The clinical features and complaints caused by sacral meningeal cysts vary from simple lumbosacral pain to a spectrum of symptoms including local pain, bowel/bladder/sex dysfunction and S1 radicular symptoms. There exists controversy regarding the efficacy of surgery in the management of sciatica. Tanaka [7] believed that since sciatica, as with low back pain, is a nonspecific symptom of the sacral cyst, thus a patient with sciatica has little chance to be cured by surgery. However, it has been reported that sciatica and intermittent claudication were relieved following surgery in the vast majority of cases [5, 6, 17, 18]. Moreover, in our series, 13 of the 16 patients with sciatica experienced abatement of the symptom postoperatively.

It is our opinion that whether or not to have the patient treated surgically depends on whether there are neurological symptoms related to the cyst. S1 radicular symptoms, perineal/perianal pain and bowel/bladder/sex dysfunction are all caused by compression of the sacral plexus, on condition that tumor, disc herniation and the like have been ruled out and the patient fails to respond to conservative treatment, then an operation intended for sacral cyst is justified. When there were multiple cysts in one patient, although some smaller cysts were not directly related to the symptoms, we tended to treat every cyst because these smaller cysts had a large chance to expand and cause symptoms in the future due to their inherent pathophysiology (e.g. case 12).

### Surgical technique

Various strategies have been proposed to manage patients with sacral cysts, but up to now, there has not been a universally accepted one. The widely accepted strategies fall into three categories: 1. Approaches to equalize the CSF pressure between the cyst and the thecal sac [8, 19, 20]. 2. Percutaneous computed tomography-guided aspiration of the cyst with/without fibrin glue placement [9, 21, 22]. 3. Surgical removal or imbrications of the cyst wall with/without communicating hole repair [4–7, 18, 23, 24]. Each of them could achieve varying degrees of symptom relief but are inevitably associated with various complications and recurrence.

Among these popular methods, no effective blockage of the communicating hole was done [6, 7, 16]. Because the cyst cavity is not obliterated and the communicating hole is not blocked, it is reasonable to worry about a recurrence of the cyst in these methods. For blocking the communication, ligation placed at the neck portion of the cyst may cause entrapment of the nerves. Alternatively, blocking the communication using a small muscle graft is the treatment of choice because it is simple and effective. Elsawaf et al. [23] tried to use a local fat graft and gelatin sponge to reinforce closed cyst neck. However, this weak reinforcement was likely to fail after the degradation of the fat and gelatin sponge. Instead, a muscle graft with firm suture would provide persistent obstruction, as confirmed by case 12. The key point of the obstruction was the elasticity of the muscle graft. The nerve root was covered by the muscle graft, which was stronger than the fat but softer than the string suture, and the sutures tied the muscle graft and the surrounding tissue instead of directly ligating the nerve root. Therefore, the obstruction could be achieved without hard and direct compression on the nerve root, which was a common problem in direct ligation techniques, and the constriction of the nerve root could be avoided. Besides, the success of obstruction could be verified by the absence of intra-operative cerebrospinal fluid flow and postoperative cerebrospinal fluid leakage.

Moreover, it is unnecessary to eliminate the cyst wall altogether since it has no excretory function. To obviate the risk of damaging the nerves, during the operation we did not give any try to remove the cyst wall forcibly but left it undisturbed. The overall satisfying outcome and relatively less recurrence and complications exhibited the safety and efficacy of our strategy.

Adjuvant procedures were applied to enhance the obstruction. After the obstruction with muscle graft, the residual space was filled with gelfoam and/or fibrin glue, or a muscle flap. Such materials were not expected to fill the cavity effectively or consistently but served as temporary compression to equalize the pressure between the cavity and the dural sac. This temporary equalization might help to prevent laceration of the muscle graft and provide a stable condition for fixation of the obstruction, thus preventing recurrence. However, the confirmation of such an assumption demands histological evidence and further study. In addition, a closed non-pressure drainage was inserted into the cavity to drain the hemorrhage in the surgical site. Furthermore, if CSF leakage happened, although not seen in the current series, the leakage could be identified immediately in the drainage and managed in time. Altogether, we would like to suggest the current strategy for potential consideration by surgeons confronting a sacral meningeal cyst case.

Limitations of our study included its retrospective nature and relatively small sample size. Additionally, the subsequent changes in the muscle graft and surrounding tissues were not investigated. A prospective study with a large cohort is in need in the future, as well as histological research in the surgical site.

## Conclusions

The sacral meningeal cyst originated from a communication with the dural sac. Surgical treatment of symptomatic sacral meningeal cysts can yield a long-term resolution of the appropriately selected patient’s symptoms. Obstructing the communicating hole with a block of muscle graft is an effective and simple method to obliterate the cyst. The incised cyst wall can be left untreated instead of resected or imbricated.

## Data Availability

The datasets used and/or analyzed during the current study are available from the corresponding author on reasonable request.

## Abbreviations

CSF: Cerebrospinal fluid
MRI: Magnetic resonance imaging
